# Mechanisms of sepsis-induced acute liver injury: a comprehensive review

**DOI:** 10.3389/fcimb.2025.1504223

**Published:** 2025-02-21

**Authors:** Yongjing Guo, Wanxu Guo, Huimin Chen, Jian Sun, Yongjie Yin

**Affiliations:** ^1^ Department of Emergency and Critical Care, the Second Hospital of Jilin University, Changchun, China; ^2^ Department of Neonate, The Second Hospital of Jilin University, Changchun, China

**Keywords:** sepsis, liver injury, pathogenesis, inflammation, metabolism, ferroptosis

## Abstract

Sepsis is a severe, often life-threatening form of organ dysfunction that arises from an inappropriately regulated host response to infectious pathogen exposure. As the largest gland in the body, the liver serves as a regulatory hub for metabolic, immune, and detoxification activity. It is also an early sepsis target organ such that hepatic dysfunction is observed in 34-46% of patients with sepsis. The precise mechanisms that give rise to sepsis-induced liver injury, however, remain incompletely understood. Based on the research conducted to date, dysregulated systemic inflammation, microbial translocation, microcirculatory abnormalities, cell death, metabolic dysfunction, and liver inflammation may all contribute to the liver damage that can arise in the context of septicemia. This review was developed to provide an overview summarizing the potential mechanisms underlying sepsis-induced liver injury, informing the selection of potential targets for therapeutic intervention and providing a framework for the alleviation of patient symptoms and the improvement of prognostic outcomes.

## 1 Introduction

Sepsis is a condition characterized by unrestrained inflammatory activity and immune dysfunction resulting from an overexuberant response to infectious agents that can culminate in lethal organ damage (Cecconi et al., 2018). Sepsis is one of the leading causes of death in intensive care unit settings and a prominent source of healthcare-related expenses (Xie et al., 2020). In 2017 alone there were an estimated 48.9 million sepsis cases globally, with a 22.5% mortality rate (Rudd et al., 2020). A 2020 cross-sectional analysis performed in China confirmed that among patients in intensive care unit (ICU) settings, sepsis incidence rates can be up to 20%, and the 3-month mortality rate among affected patients was measured at 35.5% (Xie et al., 2020). Sepsis has long been a threat to public health. While extensive resources have been devoted to treating this condition, extant therapies are primarily supportive in nature and rely on antibiotic administration, hemodynamic stabilization, and supporting organs that face a risk of failing (Van Wyngene et al., 2018). No specific treatment strategy has yet been established that is capable of consistently saving the lives of patients with sepsis (Stanski and Wong, 2020).

The liver is uniquely positioned within the body from an anatomic perspective, has a rich blood supply, and is responsible for many functions including the production of cytokines, the coordination of immune activity, and the clearance of bacteria, thus serving as a hub for the control of inflammation and immunity (Kubes and Jenne, 2018). As one of the six organs assessed in the SOFA score during sepsis, paying attention to liver injury helps in the early identification and intervention of MODS. Sepsis-associated inflammation commonly damages the liver (Protzer, 2012), and abnormal liver function is evident in 34-46% of patients affected by sepsis (Yan et al., 2014). As a form of secondary liver damage, sepsis-induced liver injury (SILI) is characterized by hepatic dysfunction, liver biochemistry results outside the normal range, and a risk of liver failure (Lu et al., 2023). Liver injury can predict poor prognostic outcomes in sepsis patients (Sun Y. et al., 2020), and SILI has been established as an independent factor that can predict mortality among patients in ICU settings (Beyer et al., 2022). There is thus a clear need to further study the mechanistic basis that underlies the pathogenesis of SILI. This review thus provides a comprehensive overview of the molecular and cellular processes that give rise to SILI, with a particular focus on highlighting promising targets for therapeutic intervention that may allow for better prognostic outcomes in affected individuals.

## 2 The pathogenesis of SILI

**Figure 1:**
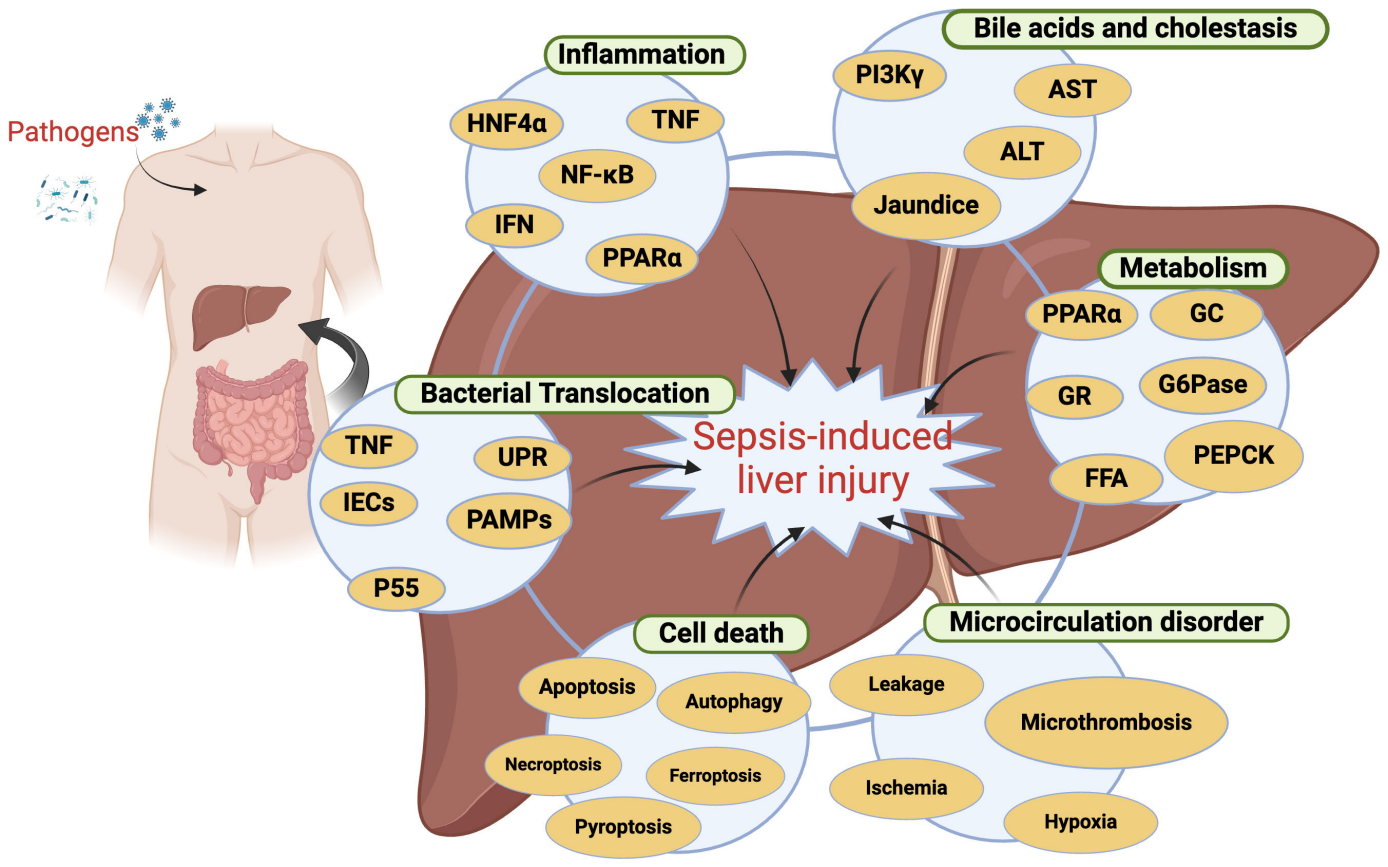
The mechanisms responsible for sepsis-induced liver injury. This image illustrates the mechanisms of liver injury caused by sepsis, highlighting the interactions between various biomarkers and processes. HNF4α, Hepatocyte Nuclear Factor 4 Alpha; TNF, Tumor Necrosis Factor; NF-κB, Nuclear Factor kappa-light-chain-enhancer of activated B cells; IFN, Interferon; PPARα, Peroxisome Proliferator-Activated Receptor Alpha; PI3K, Phosphoinositide 3-Kinase; AST, Aspartate Aminotransferase; ALT, Alanine Aminotransferase; GC, Glycogen Synthase; G6Pase, Glucose-6-Phosphatase; PEPCK, Phosphoenolpyruvate Carboxykinase; IECs, Intestinal Epithelial Cells; PAMPs, Pathogen-Associated Molecular Patterns; UPR, Unfolded Protein Response; FFA, Free Fatty Acids. Created in BioRender. Guo, Y. (2025) https://BioRender.com/n54f958.

The precise mechanistic basis for SILI has yet to be fully elucidated. While inflammation is well-established as the core foundation for sepsis-related pathology, it alone is inadequate as a means of explaining the onset of secondary organ dysfunction (Van Wyngene et al., 2018). Instead, several different processes have been established as contributors to SILI (**Figure 1**), including metabolic dysregulation, inflammation within the liver, systemic inflammation, microcirculatory abnormalities, bacterial translocation, and cell death. Each of these mechanisms can interact in a complex regulatory network, culminating in hepatic injury and the potential for liver failure.

### 2.1 Dysregulated systemic inflammatory activity

Systemic inflammation that is not appropriately regulated or directed is a hallmark of the pathogenesis of sepsis, influencing all aspects of this disorder (Manabe and Heneka, 2022). In acute sepsis, cell surface pattern recognition receptors (PRRs) found on cells such as macrophages and monocytes can recognize particular damage- or pathogen-associated molecular patterns (DAMPs and PAMPs), thereby triggering immune cell activation and the secretion of pro-inflammatory cytokines (IL-1, IL-2, IL-6, IL-8) together with the suppression of the release of IL-10 and other anti-inflammatory mediators (Beyer et al., 2022). The activation of the Toll-like receptor (TLR) and nucleotide-binding oligomerization domain (NOD) receptor families of PRRs in the context of sepsis can initially aid in the clearance of pathogens while restricting damage to tissues. When activated too strongly or for an extended period of time, however, these PRRs can trigger a cytokine storm resulting from the release of extremely high levels of inflammatory mediators, thereby causing organ failure and potentially death (Fajgenbaum and June, 2020).

As an immunoregulatory organ, the liver represents the second line of defense after the intestinal barrier as a means of preventing host invasion by pathogens (Kubes and Jenne, 2018). The liver exhibits a dual blood supply, with 75% of its blood flow coming via the portal vein, which is responsible for delivering nutrients, bacteria-derived products, and medications from the gastrointestinal tract (Ahmed et al., 2021). As a consequence of this positioning, the liver is highly susceptible to sepsis-associated inflammation and resultant hepatic dysfunction.

### 2.2 Bacterial translocation

Microorganisms can be conceptualized as the origin of higher life forms (Theis et al., 2016). Researchers have, in the past 20 years, established the concept of a so-called “holobiont”, which recognizes humans and most other animals as entities that consist of both the host itself as well as a large community of symbiotic host-associated microbes (Theis et al., 2016). This new concept has coincided with growing interest in the role that the gut microbiome plays as a regulator of human disease, Strikingly, gut bacteria have been shown to regulate immune functionality, including the sensitivity of hosts to infection-related responses (Jia et al., 2018). A recent study indicates that during sepsis, the cytokine tumor necrosis factor (TNF) disrupts the homeostatic unfolded protein response (UPR) by activating the interferon (IFN) pathway, which diminishes the production of antimicrobial peptides. This disruption leads to bacterial translocation to organs, resulting in various forms of microbial sepsis, organ failure, and death. Additionally, activation of sepsis-related genes, such as *Mmp8*, has been detected in the liver (Wallaeys et al., 2024). In this study, the authors also suggested that there may be potential therapeutic benefits from inhibiting the bacterial spread of inflammatory bowel disease using tumor necrosis factor or P55 inhibitors.

Bacterial translocation is a process wherein endogenous bacteria or products derived therefrom, including nucleic acids, phospholipid wall-derived acids, peptidoglycan components, and endotoxins can pass through the intestinal epithelium to access the mesenteric lymph nodes, systemic circulation, or extra-intestinal organs (Fouts et al., 2012). As the first site to which blood from the intestines is delivered via the portal vein, the liver is particularly sensitive to the risk of bacterial translocation. Normally, the gut lumen is surrounded by a physical barrier that consists of the mucosa and intestinal epithelial cells (IECs). This barrier layer, together with immune cells located within the intestines, normally functions to prevent the entry of bacteria or bacterial components into the portal venous system. Under septic conditions, however, dysbiosis can impact the intestinal microflora and contribute to mucus layer abnormalities, disrupted intercellular connectivity, and the apoptotic death of IECs. As a result, DAMPs and PAMPs can more readily be transported by the bile duct system and portal vein to the liver, triggering aberrant or excessive inflammation. This results in the impairment of hepatocyte functionality and compromises normal bacterial clearance while also disrupting metabolic activity (Sun J. et al., 2020).

### 2.3 Hepatic inflammation

**Figure 2:**
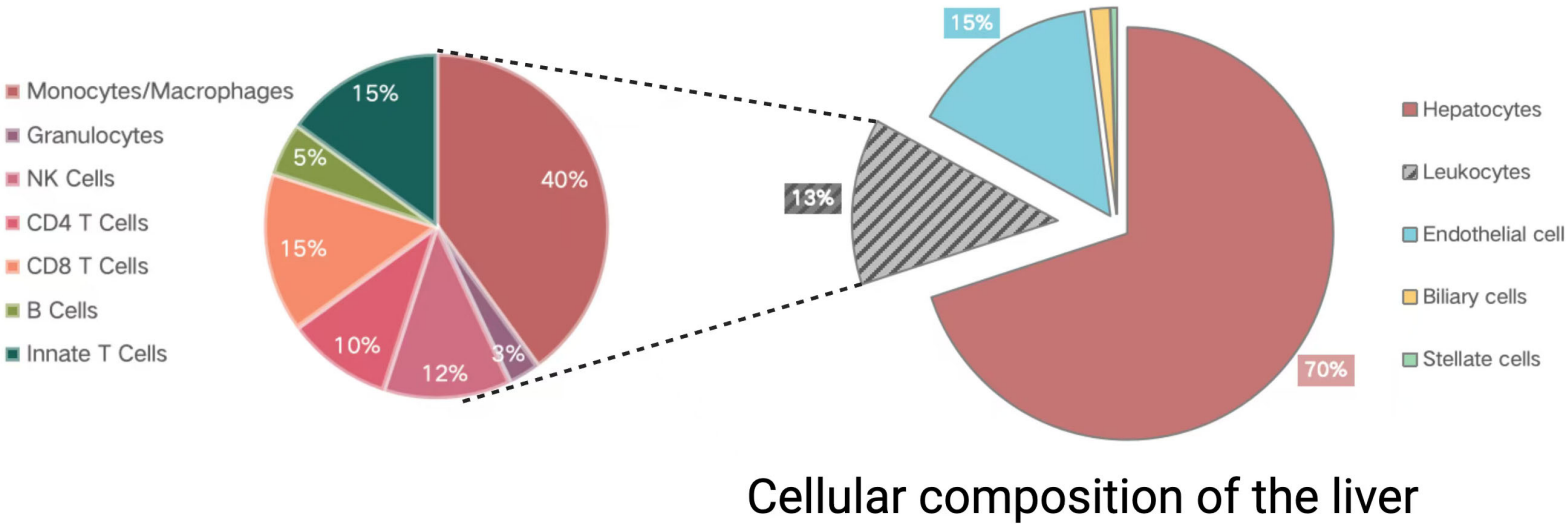
The cellular makeup of the liver. Divided into two parts: the pie chart on the left shows the composition of immune cells in the liver, while the pie chart on the right displays the main cell types in the liver. Created in BioRender. Guo, Y. (2025) https://BioRender.com/m34j126.

In addition to serving as a central regulator of immune and inflammatory responses related to sepsis, the liver is also a key target of these processes (Siore et al., 2005). The hepatic sepsis-related inflammatory response is regulated by coordination among non-parenchymal, parenchymal, and infiltrating immune cell populations. Hepatic parenchymal cells compose 70% of the liver tissue (**Figure 2**), and include both hepatocytes and cholangiocytes (Ahmed et al., 2021). Classes of non-parenchymal cells within the liver include hepatic stellate cells (HSCs), leukocytes, and liver sinusoidal endothelial cells (LSECs). Sepsis-associated immune responses can serve as a dual-edged sword capable of both eliminating bacteria and toxins derived therefrom while also triggering inflammation, suppressed immune function, and damage to end organs (Yan et al., 2014).

#### 2.3.1 The hepatic pro-inflammatory response

Inflammatory responses in patients affected by sepsis aim to clear away any pathogenic microbes. The liver is a key immune organ and a major source of inflammatory mediators. Sepsis-related impairment of intestinal function can allow gut-derived DAMPs and PAMPs to access the liver via the bile ducts and portal vein (Schnabl and Brenner, 2014). These stimuli can then trigger pathogenic bacterial clearance, leading to inappropriate immune response activity that can culminate in overwhelming inflammation (Bhogal, 2013). Research has demonstrated that simvastatin mitigates liver injury in an LPS experimental model via the NF-κB/p6/survivin signaling pathway, highlighting the protective effect of targeting inflammatory pathways in sepsis-related liver damage (Kuča, 2019).

The inner layers of the hepatic sinusoidal epithelium and capillary beds are composed of LSECs, which form a type of sieve that allows for continuous antigen monitoring and the establishment of immunological tolerance. These LSECs are also highly endocytic in nature, allowing for the uptake and metabolic degradation of small colloidal particles. LSECs can additionally present antigens to other immune cell populations, thereby controlling their activation to maintain immunological homeostasis (Gracia-Sancho et al., 2021). Seven different TLRs are expressed on the surfaces of LSECs, and bacteria-mediated activation of these receptors can trigger the initiation of an inflammatory response that coincides with the secretion of inflammatory mediators (Dalpke et al., 2006). Notably, immunoglobulin superfamily adhesion molecules that are secreted by LSECs play a key role in leukocyte migration to sites of inflammation (Knolle and Wohlleber, 2016).

Macrophages can adapt specific phenotypic characteristics during sepsis and are often broadly classified into the M1 and M2 types. M1 macrophages are pro-inflammatory in nature (Wang et al., 2021), and can be activated by LPS or other PAMPs together with Th1 cytokines such as TNF-α and IFN-γ, whereupon they secrete various cytokines that can attract activated T cells, natural killer (NK) cells, and neutrophils. These cells, in turn, release more inflammatory cytokines and reactive oxygen species (ROS) that can eliminate pathogens but can also exacerbate inflammation. When M1 polarized macrophages are persistently activated, this can result in severe hepatic injury. The liver contains one of the largest populations of macrophages in the body. As resident macrophages, Kupffer cells (KCs) are situated in the liver sinusoids, optimally positioned to sample and respond to pathogens entering the liver through the bloodstream, making them crucial regulators of pathogen defense. KCs also extend significantly into the Disse space, where they keep close contact with hepatocytes and stellate cells (Bonnardel et al., 2019), continuously monitoring the microbiota and damaged red blood cells in the hepatic sinusoids, while also participating in metabolic tasks (Guilliams and Scott, 2022). Kupffer cells and macrophages derived from infiltrating monocytes are significant sources of cytokines, such as tumor necrosis factor-alpha (TNF-α) and interleukin-6 (Tacke et al., 2009). Recent research revealed that *T. brucei brucei* infection modifies the composition of liver-resident macrophages, resulting in monocyte infiltration that differentiates into diverse infection-associated macrophage populations with unique transcriptomic profiles. Although infection-associated macrophages vanish after the infection resolves, monocyte-derived macrophages transplanted into the liver display KC-like characteristics and coexist long-term with embryonic KCs (Musrati et al., 2024). This study is the first to show that cured infections leave lasting transcriptomic and epigenomic marks on Kupffer cells, thereby altering some of their functions. Cured animals are significantly more effective at preventing secondary bacterial infections. Consequently, research into the role of KCs in the liver during infections deserves attention.

Sepsis is characterized by the hepatic accumulation of large quantities of neutrophils that can release antimicrobial proteins from their granules and can engulf pathogens (Heymann and Tacke, 2016; Protzer, 2012). They are also capable of producing neutrophil extracellular traps (NETs) with antipathogen activity. NETs form in the hepatic vascular system during sepsis (McDonald et al., 2012), and in mouse models of *Staphylococcus*-induced sepsis, NETs are capable of both eliminating hepatic bacteria while also obstructing blood flow and thereby contributing to SILI (Jenne and Kubes, 2013).

NK T (NKT) cells, which make up roughly 20% of the T cells found within the liver, comprise important mediators of antimicrobial defenses. The exposure of these NKT cells to pathogens during sepsis can trigger the release of IFN-γ, resulting in extensive neutrophil and macrophage recruitment and the promotion of hepatic damage in murine model systems (Halder Ramesh et al., 2007).

NK cells are also important regulators of sepsis-related hepatic inflammation (Souza-Fonseca-Guimaraes et al., 2013). Murine sepsis models are characterized by elevated hepatic NK cell levels, and these cells can trigger the release of cytokines including IFN-γ and TNF-α that are capable of triggering the apoptotic death of hepatocytes.

The response of hepatocytes to sepsis is known as the acute phase response, which can release various acute phase proteins (APP) with diverse biological functions into the systemic circulation. APP has multiple functions, including directly killing pathogens or coordinating the immune system to effectively eliminate them. During sepsis, hepatocytes respond to the stimulation of the cytokines in the serum such as IL-6, IL-1β, producing a significant amount of APP to combat bacteria and regulate the immune response (Zhou et al., 2016). Thus, hepatocytes can be regarded as crucial downstream effector cells actively engaged in the host immune system. Recent studies have indicated that during sepsis, the activity of hepatocyte nuclear factor 4 alpha (HNF4α) in the liver diminishes, resulting in the downregulation of peroxisome proliferator-activated receptor alpha (PPARα), metabolic complications, and dysregulation of the IL6-mediated acute phase response (Van Dender et al., 2024). In this study, hepatocyte-specific HNF4α knockout mice displayed reduced expression and activity of PPARα, diminished IL6-induced acute phase response, and increased mortality from sepsis. Notably, pre-administration of IL6 and dexamethasone maximally stimulated the acute phase response and mitigated sepsis.

In conclusion, hepatic inflammatory processes are shaped by interactions among non-parenchymal, parenchymal, and infiltrating immune cell populations within the liver, with both inflammatory factors and acute-phase proteins further shaping the associated responses.

#### 2.3.2 Hepatic immunosuppression

Immunosuppression is an important component of sepsis, presenting with dysfunctional immune activity, altered cytokine production, elevated levels of myeloid-derived suppressor cells (MDSCs), and the dysfunction of neutrophils, monocytes, and macrophages (Liu D et al., 2022). Immunosuppressive activity during sepsis is central to the incidence of secondary infections and multiorgan dysfunction (Hotchkiss et al., 2013).

Endotoxin tolerance in the liver is an important mechanism that underlies sepsis-related immunosuppression. Monocytes, for instance, can downregulate TLR4 expression on their surface such that LPS stimulation triggers less intense inflammation (Broad et al., 2006). Hepatocytes can promote cytokine signaling inhibitor 1 expression to decrease LPS sensitivity, whereas LSECs can favor LPS tolerance through reductions in nuclear NF-κB localization (Uhrig et al., 2005). The capacity of these tolerant cells to present antigens is markedly compromised, and rather than secreting pro-inflammatory cytokines they release anti-inflammatory mediators. When exposed to further endotoxins, they may thereby trigger the suppression of immune functionality.

MDSCs are cells that exhibit immune suppressive activity and influence pathological conditions including infections, chronic inflammation, cancer, and sepsis. Bone marrow-derived MDSCs can undergo hepatic recruitment during sepsis to promote immunosuppression. These MDSCs can suppress immune activity through several mechanisms, such as inhibitory cytokine secretion, the induction of T cell apoptosis, and altered immune microenvironmental activity (Gabrilovich and Nagaraj, 2009). Efforts to maintain an adequate balance between pro- and anti-inflammatory response activity may thus be vital for the amelioration of SILI.

### 2.4 Bile acids and cholestasis

Cholestasis is a frequent complication in patients with sepsis (Bhogal, 2013), encompassing both hepatocellular and ductular cholestasis. Considerable studies have been conducted on hepatocellular bile stasis, indicating that the downregulation of hepatic transport system expression, caused by pro-inflammatory cytokines and mediators, is the primary reason for inflammation-induced hepatocellular bile stasis (Geier et al., 2005) (Kosters and Karpen, 2010). Phosphatidylinositol 3-kinase gamma (PI3Kγ) is recognized as a significant trigger for sepsis-induced cholestasis through the internalization and downregulation of hepatic biotransformation by excretory organ proteins. Research has demonstrated (Recknagel et al., 2012) that PI3Kγ knockout mice are protected from experimental cholestasis induced by sepsis. Additional mechanisms include alterations in transporter protein transport, reorganization around the microtubules of the cytoskeleton, and disruption of tight junctions between hepatocytes that impact bile secretion (Strnad et al., 2017). There has been relatively limited research on the mechanisms leading to ductal cholestasis in sepsis. Cholangiocytes release inflammatory factors that promote inflammation around the ducts while simultaneously inhibiting the transport of chloride ions and bicarbonate ions in cholangiocytes, thus impairing ductal flow (Strazzabosco et al., 2005).

In a sepsis rat model, liver dysfunction is an early and common event, affecting all aspects of hepatic biotransformation, with the severity related to subsequent prognosis (Recknagel et al., 2012). The observed rise in serum bile acid concentration during cholestasis can disrupt physiological processes and adversely affect organ function. Bile acid overload results in heightened oxidative stress, increased cell membrane permeability, and hindered regeneration of bile transport proteins (Bhogal, 2013) (Liu et al., 2015),which may lead to compromised glucose and lipid metabolism, suppressed immune responses, vascular dilation, and impaired renal function (Trauner et al., 2011). Furthermore, bile acids function as hormones and activate distinct nuclear receptors that regulate intracellular metabolic pathways in hepatocytes (de Aguiar Vallim et al., 2013).

### 2.5 Metabolism

The liver is an essential metabolic organ that shapes fat and protein synthesis, blood glucose control, and overall energy metabolism (Ramos Figueira, 2015). Sepsis entails the release of significant levels of catecholamines and cortisol, which can induce a highly catabolic state that readily depletes fat, protein, and carbohydrate reserves (Lelubre and Vincent, 2018). Reversing deleterious sepsis-related metabolic changes is vital for the prevention and alleviation of SILI.

#### 2.5.1 Glucose metabolism in SILI

Glucose metabolism is a tightly regulated process (Herling et al., 2011). When meals are complete, circulating glucose is transported into cells in the liver wherein it can be processed via oxidative phosphorylation to generate energy or stored in the form of glycogen. Decreases in serum glucose levels, as occur when fasting or under starvation conditions, prompt the breakdown of glycogen to replenish these glucose levels together with increased fat and protein utilization to fuel gluconeogenesis and the production of energy. During sepsis, the liver’s non-insulin-mediated glucose uptake can lead to increased glucose utilization, potentially resulting in hypoglycemia. In this context, glycogenolysis and gluconeogenesis in the liver should serve as the primary sources for maintaining systemic glucose homeostasis. However, studies have indicated that the activity and/or expression of the rate-limiting enzyme PEPCK/G6pase in hepatic gluconeogenesis are diminished during sepsis, suggesting that the efficiency and extent of gluconeogenesis in the liver are impaired during this condition (Van Cromphaut et al., 2008).

During the infection process, the hypothalamic-pituitary-adrenal (HPA) axis is activated by pro-inflammatory mediators, resulting in the synthesis of glucocorticoids (GC). It is estimated that 10% to 20% of critically ill patients and as many as 60% of patients with septic shock demonstrate HPA axis dysfunction (Annane et al., 2017). To address the deficiency of endogenous steroids, exogenous GCs are utilized as an adjunctive treatment for sepsis. In published studies on GC treatment for septic shock, a significant correlation was observed between the delay in GC intervention and mortality, which may indicate the onset of GC resistance following sepsis; that is, after GC treatment or despite seemingly adequate plasma GC concentrations, glucocorticoid receptors (GR) exhibit insufficient response in regulating gene expression. Vandewalle conducted a genome-wide study on GC resistance in septic animal models and discovered that the rapid and progressive exhaustion of GR function causes dysregulation of gluconeogenesis in hepatocytes, leading to the accumulation of lactate in the blood (Vandewalle et al., 2021).

These inflammation- and oxidative stress-driven cascading effects can engage fibrotic signaling pathways, resulting in apoptotic cell death caused by high levels of cytokine production, thereby damaging the liver. In its late stages, sepsis can also cause hypoglycemia resulting from mechanisms such as insufficient dietary intake, the depletion of glycogen, reductions in gluconeogenesis, and disordered metabolic activity. Both hypoglycemia and hyperglycemia can harm patients and influence their clinical prognosis (Wasyluk and Zwolak, 2021).

#### 2.5.2 Lipid metabolism in SILI

Sepsis is associated with various changes in lipid metabolism, which can lead to disorders in lipid and lipoprotein metabolism. During sepsis, an overactive immune response, mitochondrial damage, and inadequate feeding can induce a state of starvation, swiftly depleting essential energy molecules like glycogen and glucose, which are replenished through lipolysis (Muniz-Santos et al., 2023). The free fatty acids (FFA) generated by lipolysis are mainly absorbed by the liver and undergo β-oxidation to produce energy and ketone bodies (Wang et al., 2023). However, under the pathological conditions of sepsis, glucocorticoid receptors, peroxisome proliferator-activated receptor (PPARα), and other critical factors in lipid metabolism become inactivated, leading to the accumulation of harmful metabolites in the liver, such as free fatty acids and lactate (Paumelle et al., 2020).

The liver is a major site of lipid metabolism, but under sepsis-related conditions it can shift its metabolic focus toward carbohydrate metabolism, leading to a reduction in fatty acid utilization. Research indicates that PPAR-α signaling is markedly diminished in patients with sepsis and shows a positive correlation with systemic inflammation markers. Furthermore, systemic Pparα knockout mice demonstrate heightened mortality following bacterial infection (Paumelle et al., 2020).In animal studies, LPS has been shown to reduce the levels of both PPAR-α and PPAR‐y co‐activator (PGC)‐1α (Van Wyngene et al., 2018), contributing to the disruption of β-oxidation and the incidence of lipotoxicity, resulting in further liver damage.

Hepatic fat accumulation has been reported in sepsis patients (Koskinas et al., 2008). When these lipids accumulate, non-oxidative pathways can trigger the production of toxic lipid-derived substances, contributing to mitochondrial damage, abnormal signal transduction, and increases in cell death that cause further injury to target organs (Fang et al., 2022).

Mechanistic interactions between hepatic metabolism and damage can result in several pathological processes that include fibrosis, hepatocellular stress, inflammation, and metabolic dysregulation. The complex and interrelated mechanisms highlight the need to analyze abnormal lipid metabolism and to implement appropriate hepatoprotective strategies when managing sepsis.

#### 2.5.3 Amino acid metabolism

Neuroendocrine changes can develop from sepsis-related inflammation, and human metabolism can be additionally modulated by inflammatory cytokines (Di Girolamo et al., 2017). This results in a shift away from normal metabolic activity towards a state of hypercatabolism that may initiate or exacerbate organ damage. In the early stages of sepsis, enhanced hepatic amino acid uptake supports gluconeogenesis, which is crucial for meeting the liver’s energy and oxygen demands. However, when sepsis-related liver dysfunction arises, the liver’s capacity to uptake amino acids diminishes. Changes in plasma or serum amino acids are closely linked to liver injury and inflammatory responses. Research shows that alanine can disrupt the osmotic barrier and enhance the uptake of aminoglycoside antibiotics to eliminate antibiotic-resistant strains (Peng et al., 2015) and plays a crucial role in cytokine production (Chu et al., 2021). Experimental ALA supplementation in sepsis may further enhance sepsis outcomes.

### 2.6 Microcirculatory dysfunction

Sepsis is characterized by a pronounced increase in endothelial permeability. While this may initially provide a benefit to the function of host immunity as it can allow antimicrobial peptides and antibodies to pass across the endothelium, over time it can give way to microcirculatory dysfunction (Colbert and Schmidt, 2016).Microcirculatory alterations are commonly observed in the context of sepsis (De Backer et al., 2002; Simkiene et al., 2020). Such microcirculatory dysfunction plays a key role in the development of organ failure in the context of sepsis, including SILI and associated liver dysfunction.

**Figure 3:**
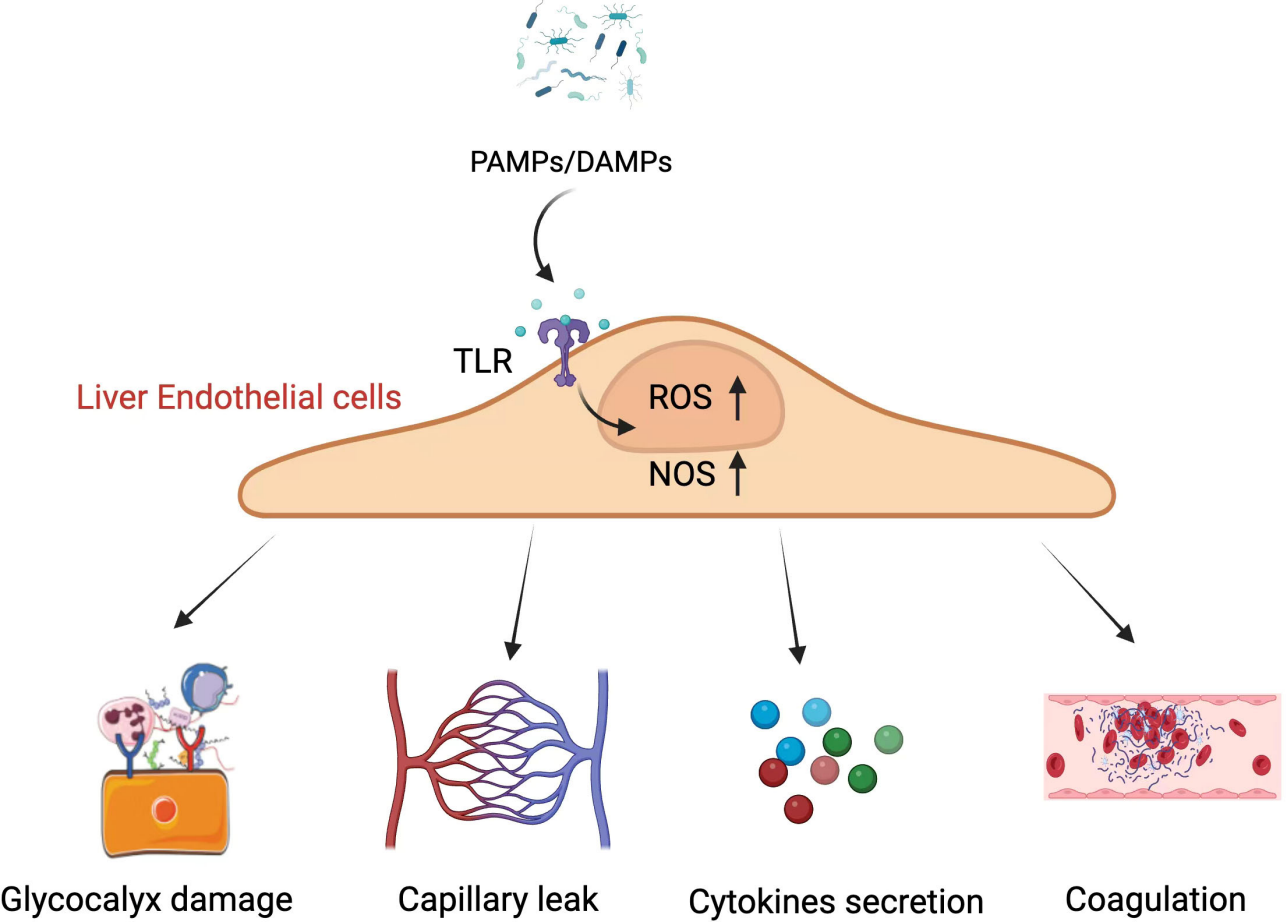
The mechanistic basis of microcirculatory dysfunction involves PAMPs and DAMPs acting on liver endothelial cells, which leads to an increase in intracellular ROS and NOS, resulting in a cascade of pathological changes. PAMPs, Pathogen-Associated Molecular Patterns; DAMPs, Damage-Associated Molecular Patterns; TLR, Toll-Like Receptor; ROS, Reactive Oxygen Species; NOS, Nitric Oxide Synthase. Created in BioRender. Guo, Y. (2025) https://BioRender.com/t04g942. The image "Glycocalyx damage" in this figure is cited from the article "Oxidative Stress and Endothelial Dysfunction in Sepsis and Acute Inflammation," authored by Joffre and Hellman, 2021. This Open Access article is distributed under the terms of the Creative Commons License [CC-BY] (http://creativecommons.org/licenses/by/4.0).

As sepsis develops, several different mechanisms can disrupt hepatic microcirulatory function (**Figure 3**). For instance, hepatic endothelial cells can be exposed to DAMPs or PAMPs transported via the bile duct system or portal vein into the liver, thereby triggering their activation and phenotypic reprogramming toward a more inflammatory, adhesive, and pro-coagulant state (Colbert and Schmidt, 2016). Sepsis can also harm the endothelial glycocalyx and trigger endothelial cell apoptosis, altering endothelial permeability and contributing to capillary leakage and perfusion-related injury in the liver (Ait-Oufella et al., 2010). Impaired microcirculatory activity leads to the emergence of areas with poor tissue perfusion that are subject to hypoxic damage (De Backer et al., 2006), and mitigating this microvascular dysfunction wherever possible can lower lactate levels and improve overall hepatic functionality (Trzeciak et al., 2008). These microcirculatory changes are thus a key facet of the pathogenesis of SILI while also being viable targets for therapeutic intervention.

### 2.7 Cell death

The death of hepatocytes is central to the development of SILI, as these dead and dying cells release DAMPs and fragmentary debris that can directly and indirectly trigger hepatic inflammation and further injure hepatocytes. Several types of cell death have been demonstrated to contribute to SILI pathogenesis.

#### 2.7.1 Apoptosis

Apoptosis is a meticulously regulated form of programmed cell death, commonly known as “cellular suicide.” It plays a vital role in an organism’s development, tissue homeostasis, and response to injury. Recent research suggests that apoptosis is triggered by the activation of Bax and the cleavage of Caspase 3 protein, alongside the inhibition of Bcl-2 protein expression. A research of shown (Alaaeldin et al., 2024). that vincristine upregulates bcl2 protein expression while downregulating bax and cleaved caspase 3 protein expression in a CLP-induced mouse model, significantly improving liver histological abnormalities and reducing liver enzymes (ALT and AST). In LPS-induced mouse models of liver and hepatocellular septic injury, elevated levels of hepatocyte apoptosis have been observed, and inhibiting apoptosis can mitigate LPS-induced septic liver damage (Cui et al., 2023). Therefore, regulating liver apoptosis during sepsis may represent an effective therapeutic target.

#### 2.7.2 Necroptosis

Necroptosis is a recently identified form of programmed cell death that displays features of both necrosis and apoptosis, and is tightly regulated by receptor-interacting protein kinases (RIP) 1 and 3 (Sai et al., 2019). Previous research has shown that necroptosis in endothelial cells is crucial to the systemic inflammatory response syndrome, indicating that modulation of necroptosis could offer clinical benefits for patients with sepsis (Zelic et al., 2018). Traditionally, apoptosis and necrosis have been regarded as the two primary modes of cell death contributing to the pathogenesis of liver diseases. Recently, necroptosis has emerged as a novel mode of cell death in sepsis-related liver injury (Zhang et al., 2024). In an LPS-induced piglet sepsis model, necroptosis was observed in the liver, and pretreatment with the specific necroptosis inhibitor necrostatin-1 significantly decreased markers of necroptosis and resulted in the alleviation of liver inflammation and injury (Xu et al., 2021). In-depth research into necroptosis during sepsis-related liver injury may be crucial in reducing liver damage caused by sepsis.

#### 2.7.3 Autophagy

Autophagy is a dynamic, lysosome-dependent, conserved metabolic process that manages damaged organelles, metabolizes macromolecules, and eliminates intracellular microorganisms to maintain cellular homeostasis (Levine and Kroemer, 2019). However, during sepsis, the dysregulation of autophagy can result in hepatocyte injury. Autophagy also coordinates macrophage polarization, inflammasome activation, and the release of pro-inflammatory cytokines, contributing to tissue damage during sepsis (Deretic, 2022). Research has demonstrated that in the livers of CLP-induced sepsis mouse models, there is an increased accumulation of autophagy protein-II and SQSTM1 (p62) (Fang, 2023). By enhancing lysosomal function and the expression of autophagy-related genes, the inflammatory response can be mitigated, potentially preventing organ damage in sepsis. Another study also indicates that carbamazepine may prevent septic liver injury by fully activating autophagy, providing new targets for the treatment of septic liver damage (Lin et al., 2014).

#### 2.7.4 Ferroptosis

Ferroptosis is a distinct form of cell death associated with glutathione depletion and extensive lipid peroxidation that occurs in an iron-dependent fashion, ultimately leading to ROS biogenesis and cell death (Shojaie et al., 2020). Many studies have highlighted the role that ferroptosis plays in the pathogenesis of SILI. In CLP-induced murine sepsis model systems, ferroptotic activity has been observed as evidenced by increases in hepatic iron levels, increases in MDA accumulation, and GSH depletion (Wang et al., 2022). Decreases in hepatic SLC7A11 and GPX4 epression were observed in a murine LPS-induced sepsis model, together with higher levels of MDA and a reduction in SOD and GSH levels. Iron suppressor-1 administration was sufficient to reverse these effects and to obviate liver injury in these sepsis model animals (Xie et al., 2023). Knocking down YAP1 in CLP-induced sepsis model mice was sufficient to exacerbate the ferroptotic death of hepatocytes and associated liver injury, while YAP1 overexpression *in vitro* within hepatocytes was sufficient to mitigate LPS-induced ferroptotic death (Wang et al., 2022). Ferroptosis thus appears to be an important contributor to SILI pathogenesis, and efforts to inhibit ferroptosis may represent an important strategy for the prevention of hepatic injury in patients suffering from sepsis. However, some studies have indicated that pharmacological and genetic approaches to inhibit ferroptosis do not enhance outcomes in sepsis and septic shock (Van Coillie et al., 2022). Consequently, the role of ferroptosis in septic liver injury necessitates further investigation, which could be a potential avenue for improving the prognosis of patients with septic liver injury.

#### 2.7.5 Pyroptosis

As a form of programmed cell death, pyroptosis is defined by the regulatory role of gasdermin proteins and the induction of the condensation, random fragmentation, and degradation of the DNA that leads to the swelling and lysis of affected cells (Jiang et al., 2023).As the primary executors of pyroptosis, gasdermins function by generating pores in the cell membrane (Wen et al., 2022). The two primary pathways of pyroptotic induction include the inflammasome-mediated and atypical processes, both of which are relevant in the pathogenesis of sepsis (Chen et al., 2016; Jiang et al., 2023). Early pyroptotic activity can facilitate pathogen clearance, as associated innate immune and inflammatory activity can disrupt intracellular pathogen replication and engage mechanisms that phagocytose and kill the causative microorganisms. When not appropriately regulated, however, pyroptosis can lead to the exacerbation of inflammatory activity within cells and tissues, leading to more severe damage that has the potential to culminate in multiple organ failure. Elevated caspase-1 and NLRP3 expression have been reported in a CLP-induced model of murine sepsis, with the inhibition of pyroptosis alleviating liver damage in these animals (Chen, 2023). In this same model system, peroxisome proliferator-activated receptor γ activity can abrogate ROS accumulation and suppress TXNIP/NLRP3 pathway signaling activity, thereby decreasing the induction of pyroptotic death and limiting the degree of hepatic injury (Li et al., 2022).Currently, several drugs have been shown to alleviate SLI by inhibiting the NLRP3 inflammasome, such as cinnamon and theaflavins (Chen, 2023; Lee et al., 2015). Pyroptosis is thus a relevant contributing factor in SILI and may serve as a promising target for therapeutic intervention.

## 3 Biomarkers of SILI

Liver dysfunction is both a risk factor for sepsis and a cause of multiple organ failure. Liver injury, especially in the context of septic shock, significantly increases the mortality rate of patients with sepsis. SILI typically involves various biomarkers used to assess liver damage and the extent of injury. We categorize the biomarkers of SILI into two main types: traditional biomarkers and presumed biomarkers.

### 3.1 Traditional biomarkers

Traditional biomarkers include albumin, bilirubin, ALT, AST, and GGT. Albumin is a major plasma protein synthesized by the liver, playing roles in maintaining colloid osmotic pressure, transport, and antioxidant activity. A decrease in albumin levels typically indicates impaired hepatic synthetic capacity and can help assess the extent of liver damage. Bilirubin is a yellow pigment produced after the breakdown of red blood cells, primarily metabolized and excreted by the liver. Liver damage caused by sepsis can lead to impaired bilirubin metabolism and excretion, resulting in elevated bilirubin levels. Additionally, liver enzymes play a crucial role in liver injury; ALT is mainly found in the liver and reflects the degree of liver damage, while AST is present in the liver, heart, muscles, and kidneys, with elevated levels usually associated with hepatocellular injury, but can also rise due to cardiac or muscle injury. GGT is related to liver and biliary function. In clinical practice, these biomarkers are often used in combination for a comprehensive assessment of liver function.

It is important to note that traditional biomarkers have certain limitations. Firstly, many traditional markers lack liver specificity. For example, AST and ALT are not only present in the liver but also in the heart, muscles, and other tissues. Furthermore, the elevation of liver enzymes can be caused by various factors, including medications, alcohol, infections, and metabolic diseases, making it difficult to determine the specific cause. Secondly, there is a lack of sensitivity, as liver damage may occur before the elevation of liver enzyme levels, hindering early diagnosis and assessment. Lastly, individual differences, such as age, sex, and ethnicity, must also be considered. Therefore, there is a need for research into emerging biomarkers.

### 3.2 Putative biomarkers

Emerging liver injury biomarkers are increasingly used in the drug development process to detect hepatotoxicity, enhancing the diagnostic and prognostic capabilities of traditional biomarkers. In this section, we summarize several of these biomarkers.

Arginase-1 (ARG1) is a cytosolic enzyme that is constitutively expressed throughout the liver. ARG1 is a member of the urea cycle, driving collagen synthesis and cell proliferation. In studies related to liver diseases, ARG1 can regulate nitric oxide levels and vascular function (Durante et al., 2007), modulate immune responses, and promote tissue repair (Wynn and Ramalingam, 2012).

Malate dehydrogenase-1 (MDH1) is expressed in the liver, but it is also found in the heart, skeletal muscle, kidneys, and spleen. MDH1 is involved in the tricarboxylic acid cycle and the malate-aspartate shuttle, playing a crucial role in mitochondrial NADH supply for oxidative phosphorylation (Shi et al., 2024). Research indicates that MDH1 deacetylation appears to promote acute liver failure by regulating NETosis (Wang et al., 2024).

α-Glutathione S-transferase (α-GST) is a member of a family of enzymes that contain distinct subunits B1 and B2 and make up approximately 3% of cytosolic protein in the hepatocyte. α-GST catalyzes the binding of reduced glutathione to exogenous substrates for detoxification purposes. Studies have shown that elevated levels of α-GST occur earlier than increases in plasma lactate and liver transaminases, suggesting that α-GST may be a more sensitive indicator of early liver injury, applicable for monitoring hepatocellular damage during the progression of sepsis (Maina et al., 2016). Additionally, research has indicated that α-GST serves as a biomarker for liver injury during sepsis caused by various microorganisms (Maina et al., 2016),demonstrating greater characteristic significance than traditional biomarkers such as GGT and albumin.

5’-nucleotidase (5-NT) is an enzyme that catalyzes the cleavage of 5’ nucleotides (dephosphorylation of nucleotides to nucleosides) and is highly expressed in the hepatocyte plasma membrane. Studies have shown that compared to healthy controls, serum 5’-nucleotidase levels are on average three times higher in viral hepatitis, 2.5 times higher in alcoholic liver disease, and two times higher in cirrhosis (Hyder, 2016). Therefore, the detection of 5-NT has certain clinical value in the differential diagnosis of hepatobiliary diseases and in assessing the extent of lesions.

Sorbitol dehydrogenase (SDH) is highly expressed in the liver and is an enzyme that regulates carbohydrate metabolism. Studies have shown that SDH expression is elevated in ischemia-reperfusion mice, and administering a sorbitol dehydrogenase inhibitor (SDI) to these mice can significantly alleviate liver injury induced by ischemia-reperfusion (Zhang et al., 2015). Additionally, other research indicates that elevated preoperative SDH levels are associated with poor postoperative prognosis in patients with hepatocellular carcinoma (Jeon et al., 2021).

Non-coding RNA (ncRNA) is a class of RNA molecules that are not translated into proteins and are highly conserved throughout evolution. They are crucial for chromatin modification, transcription regulation, and post-transcriptional processing(Wang et al., 2025). ncRNA can be categorized based on their length into short ncRNA, intermediate ncRNA, and long non-coding RNA (lncRNA). Short ncRNA includes small interfering RNA (siRNA), microRNA (miRNA), and PIWI-interacting RNA. Research has shown that ncRNAs, including miRNAs, lncRNAs, and circRNAs, promote or inhibit the occurrence and development of SILI by regulating inflammatory responses, oxidative stress, mitochondrial function, and programmed cell death. Furthermore, advancements in sequencing and detection technologies have made it possible to accurately quantify ncRNA expression levels, paving the way for the development of non-invasive diagnostic tools for early detection and monitoring of SILI (Wang et al., 2025).

## 4 Conclusion

Sepsis-induced liver damage remains an important predictor of adverse prognostic outcomes in patients suffering from sepsis. The mechanisms of septic liver injury are complex, involving various biological processes and signaling pathways, and are the result of the interaction of multiple factors. Sepsis leads to the release of a large number of inflammatory mediators, which can affect the metabolic function of liver cells, resulting in elevated liver enzymes, while also inducing programmed cell death in hepatocytes. Additionally, metabolic disorders in liver cells, particularly lipid metabolism disorders, can amplify the inflammatory response and induce programmed cell death. With a significant amount of cell death, intracellular inflammatory mediators can be released, activating the immune response of surrounding cells and further exacerbating inflammation. Thus, it is evident that there is a complex interplay between different mechanisms. Efforts to more fully document the pathogenic mechanisms that underlie SILI are vital for the development of more efficacious interventional strategies.

In this review, we discuss several genes that are currently the focus of research into various mechanisms and are potential targets for future studies on septic liver injury. Early studies identified TNF-α and NF-κB as the two most common molecules associated with the pathogenesis of SILI. TNF-α is an inflammation-related cytokine produced by monocytes and macrophages, capable of inducing cell necrosis or apoptosis through various intracellular signaling mechanisms (Alshevskaya et al., 2024). NF-κB is a crucial intracellular nuclear transcription factor that can be activated by LPS to enter the nucleus and function as a transcription factor, regulating the transcription of various genes that encode pro-inflammatory cytokines, adhesion molecules, and chemokines (Baldwin, 1996). In recent years, research has increasingly focused on fundamental studies. During this period, the NLRP3 inflammasome, PPARα, and HNF4α have become significant subjects of research. The NLRP3 inflammasome forms as a protein complex in response to cellular disturbances, resulting in inflammatory cell death (pyroptosis). In sepsis, excessive inflammasome activation and cellular pyroptosis are linked to liver injury (Coll et al., 2015).PPARα is a transcription factor crucial for the catabolism of free fatty acids; during sepsis, diminished expression of PPARα results in the accumulation of free fatty acids in the blood and liver, leading to cytotoxicity (Vandewalle et al., 2022). Recent studies indicate that HNF4α serves as a crucial activator of *Ppara* mRNA expression in the liver by binding to the DR1 motif within the PPARα gene promoter, which is vital for sustaining healthy liver metabolism, acute-phase responses, and liver regeneration (Van Dender et al., 2024).

This review provides a comprehensive foundation for researchers seeking to understand the key factors that underlie SILI, which include hepatic inflammation, microbial translocation, dysregulated systemic inflammatory activity, cell death, metabolic abnormalities, and microcirculatory dysfunction. This article thus aims to serve as a resource to support further research focused on preventing and treating SILI.
